# Extracting hierarchical features of cultural variation using network-based clustering

**DOI:** 10.1017/ehs.2022.15

**Published:** 2022-05-02

**Authors:** Xiran Liu, Noah A. Rosenberg, Gili Greenbaum

**Affiliations:** 1 Institute for Computational and Mathematical Engineering, Stanford University, Stanford, California, USA; 2 Department of Biology, Stanford University, Stanford, California, USA; 3 Department of Ecology, Evolution and Behavior, The Hebrew University of Jerusalem, Jerusalem, Israel

**Keywords:** Cultural evolution, hierarchical clustering, network

## Abstract

High-dimensional datasets on cultural characters contribute to uncovering insights about factors that influence cultural evolution. Because cultural variation in part reflects descent processes with a hierarchical structure – including the descent of populations and vertical transmission of cultural traits – methods designed for hierarchically structured data have potential to find applications in the analysis of cultural variation. We adapt a network-based hierarchical clustering method for use in analysing cultural variation. Given a set of entities, the method constructs a similarity network, hierarchically depicting community structure among them. We illustrate the approach using four datasets: pronunciation variation in the US mid-Atlantic region, folklore variation in worldwide cultures, phonemic variation across worldwide languages and temporal variation in first names in the US. In these examples, the method provides insights into processes that affect cultural variation, uncovering geographic and other influences on observed patterns and cultural characters that make important contributions to them.

## Introduction

In recent years, increasingly available large-scale datasets on aspects of variation across human cultures and within cultures over time have provided rich information about fine-scale details of human cultural variation and the factors that influence its dynamics (Mesoudi, 2016; Kolodny et al., 2018). For example, investigations of variation in folktales among cultures have identified interactions of cultural diffusion and demic diffusion in the spread of folklore and mythology (Bortolini et al., 2017; Thuillard et al., 2018). A study of design features of traditional canoes across Polynesian societies has suggested a faster rate of cultural change in canoe traits that were less significant to functional performance of the watercraft, in line with the faster evolution that occurs for non-functional rather than functional genetic variants (Rogers & Ehrlich, 2008). Studies of variation in the presence and absence of linguistic characters across languages have uncovered influences of ancient migrations on patterns of language variation observed today (Atkinson, 2011; Creanza et al., 2015).

The analysis of complex data to reveal features of cultural variation makes use of a variety of statistical methods designed for high-dimensional data analysis more generally. Such methods include analyses of distance matrices based on cultural traits of interest (Rogers & Ehrlich, 2008; Creanza et al., 2015; Bortolini et al., 2017; Thuillard et al., 2018), multivariate analysis techniques such as principal components analysis (Creanza et al., 2015), correlations involving spatial statistics and geographic maps (Atkinson, 2011; Creanza et al., 2015; Bortolini et al., 2017) and hierarchical tree-based clustering (Creanza et al., 2015; Thuillard et al., 2018).

Viewed in relation to their underlying generative processes, different forms of cultural variation often possess shared features (Cavalli-Sforza & Feldman, 1981; Boyd & Richerson, 1985). Different cultural entities might possess a shared variant, as a result of processes such as the independent origin of functionally significant variants, random recurrence of non-functional variants or cultural exchange. Salient among the forces contributing to patterns of cultural variation is shared descent, so that even if independent origins and cultural exchange are important in specific settings, hierarchical or geographic structure can often contribute to features of cultural variation.

Owing to the importance of shared descent in influencing cultural variation, tools for analysing cultural variation data can employ methods suited to the analysis of genetic data, which also possess signatures of shared descent; thus, many statistical methods used in cultural data analysis are similar to those used for genetic data (Bromham, 2017; Gray et al., 2010; Pagel, 2009). Recently, we have introduced a method, *NetStruct*, for use in understanding genetic variation data that result from hierarchical genetic structure (Greenbaum et al., 2016, 2019). The method, employing ideas from network analysis, produces a distinctive form of visualization of hierarchical population relationships. It has been seen to detect subtle patterns that have been overlooked using earlier forms of data analysis.

The *NetStruct* method consists of three main steps: construction of similarity matrices between entities; community detection in similarity matrices; and hierarchical visualization of communities. The method is general beyond genetic data, as the form of the data contributes only to the choice of similarity function. It can thus be modified for use with other types of data that result from distinct but related generative processes, including data on cultural variation.

Here, we adapt the *NetStruct* method for use in the study of cultural evolution. We examine a variety of datasets on different forms of cultural variation, considering geographic variation in English pronunciation, variation across cultures in folklore, phonemic variation across languages and temporal variation in frequencies of first names. Using each of the four forms of cultural data, we illustrate the potential of the method as an exploratory tool to reveal features of geographic and temporal structure in cultural phenomena and to extract patterns that can inspire hypotheses about underlying mechanisms. Each example additionally highlights a different aspect of the hierarchical analysis: analyses at different levels of detail in the hierarchy, identification of characters that are important in driving the partitioning, analysis of outliers and the relationship of the hierarchy to features of entities beyond those used in its construction.

## Results

### *Generalizing the* NetStruct *pipeline*

In the first step of the *NetStruct* method, for a set of entities, each having a value for each of a series of characters, we construct an *n* × *m* data matrix *A* with *n* rows corresponding to entities and *m* columns corresponding to characters. Entry *A*_*ij*_ gives the value of character *j* for entity *i*; this value can be either categorical or quantitative, depending on the type of character.

The similarity between two entities *i*_1_ and *i*_2_, denoted *s*_*i*1,*i*2_, is computed by a function applied to rows *i*_1_ and *i*_2_. We normalize pairwise similarities so that they take on values in [0,1]. The resulting *n* × *n* similarity matrix *S* then becomes the adjacency matrix of a similarity network. The similarity function is chosen based on a particular application of interest.

In a network, community structure exists when high concentrations of edges occur within certain groups of nodes in the network and low concentrations occur between these groups (Girvan & Newman, 2002). In the second step of *NetStruct*, we iteratively remove edges with lower weights from the network to reveal the finer-scale structure within coarser communities. *NetStruct* uses a community-detection Louvain algorithm (Blondel et al., 2008) together with an iterative edge-pruning method (Greenbaum et al., 2019). The Louvain algorithm maximizes a ‘modularity score’ for each community, quantifying the difference between the actual density of edges within the community and the expected density if all edges in the network were distributed at random while preserving the degree distribution of the network. The Louvain algorithm starts by assigning each node to its own community, sequentially merging nodes into communities in a manner that produces the greatest modularity increase – until no further increase occurs. *NetStruct* iteratively removes edges below a weight threshold of increasing value and applies community detection in each subdivided community at each iteration, generating hierarchical structure at multiple levels.

Finally, in the last step, the communities detected at each iteration are assembled to form the output hierarchy, which can be visualized as a hierarchical tree coded by a colouring scheme. Because clustering is hierarchical, each entity can belong to multiple communities, or clusters, at different hierarchical levels; that is, each cluster can have finer-scale ‘child’ clusters.

*NetStruct* visualizes community structure using a diagram that depicts hierarchical relationships among clusters. Each cluster is assigned an interval representation of a colour gradient; the root node is assigned the unit interval. Child clusters are assigned equal portions of the interval associated with their parental node. Clusters are coloured by the midpoint of the associated interval, such that at each hierarchical level, child clusters of the same parent have colours that are more similar than are those of different parents. The colour scheme facilitates interpretation, as the original entities can be labelled by the finest-scale cluster to which they are assigned in the diagram.

To generalize the use of *NetStruct* beyond genetic data, we require a function that describes similarity between pairs of entities of interest. Many similarity measures are possible, and *NetStruct* is applied to the similarity matrix after it has been constructed. For a given dataset of interest, the similarity function is chosen in a manner suited to the application. We follow Greenbaum et al. (2016, 2019) in choosing frequency-weighted similarity measures that emphasize shared rare values of a character.

### Variation in pronunciation across locations

For our first example, we examined data on individual variation in pronunciation. Local variation in communication variants has the potential to provide insight into cultural transmission and spatial patterns of distinctiveness and interaction in a population (Nerbonne & Kleiweg, 2003; Rendell & Whitehead, 2005; Aplin, 2019). To understand the relationship between geography and individual-level pronunciation of a shared human language, we applied *NetStruct* to data on English pronunciation variation in the middle and south Atlantic region of the US.

#### LAMSAS pronunciation data

We obtained pronunciation variation data from the Linguistic Atlas of the Middle and South Atlantic States (LAMSAS; Kretzschmar et al., 1993). These data consist of dialect records on pronunciations of everyday words collected in 1933–1942 from 11 states: Delaware, Maryland, New Jersey, New York, North Carolina, Pennsylvania, South Carolina, Virginia and West Virginia, with some records from eastern Georgia and northeastern Florida included as well.

We restricted our analysis to *n* = 839 informants interviewed by the major field worker (Nerbonne & Kretzschmar, 2003) and *m* = 69 words recorded for most informants. We constructed the *n* × *n* similarity matrix based on phonetic transcriptions of pronunciations of the *m* words. The similarity is greater when informants share many pronunciations, and when they share rare pronunciations (see the Methods section). We then applied *NetStruct* to infer hierarchical structure.

#### Hierarchical structure of pronunciation variation: Levels of detail

**Figure 1.**
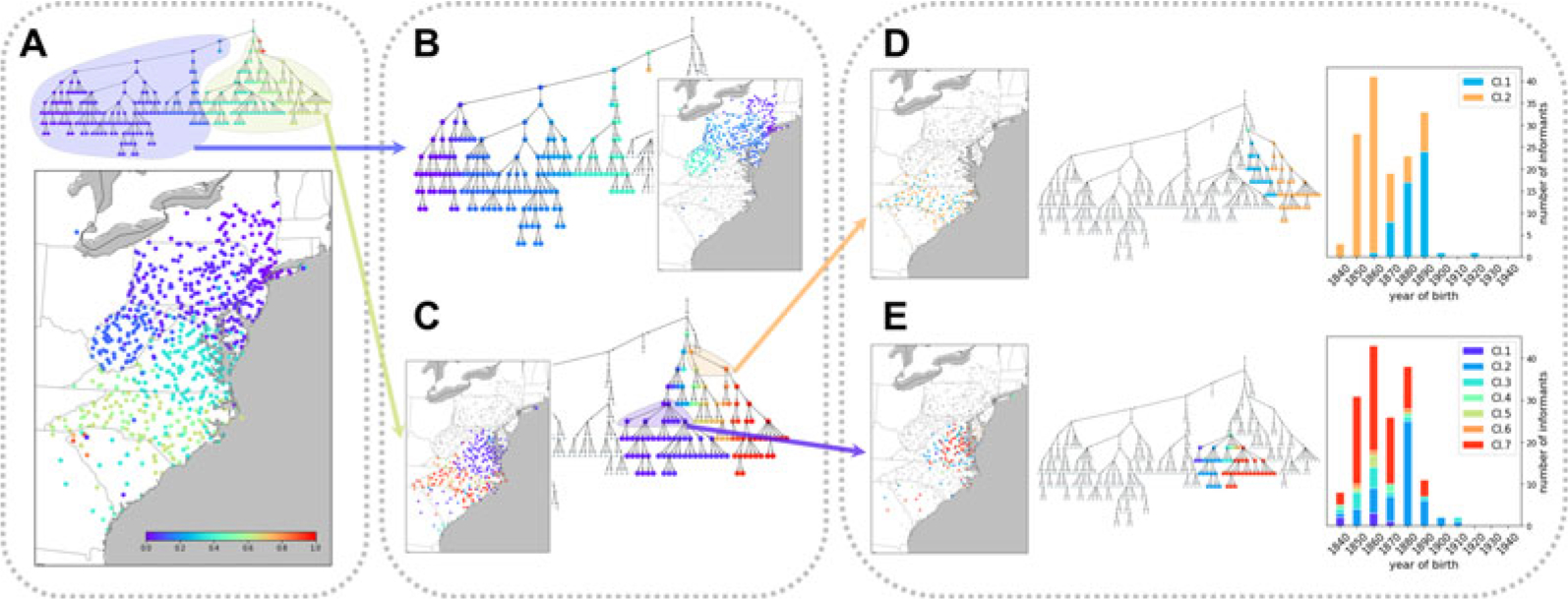
Hierarchical features of variation in English pronunciation in the middle and south Atlantic region of the US. (a) Hierarchical tree of the pronunciation similarity network. Informants are marked on the map by the colour of the finest-scale cluster to which they belong. (b, c) Two major clusters detected at the first level of the hierarchy in (a), each re-coloured with the full colour interval. (d, e) Two finer-scale clusters of the hierarchy in (c). In these panels, colours are assigned based on placement in the area of the hierarchy circled in (c), with all descendants of a child in the circled area assigned the same colour. The colours in (d) correspond to 1/4 and 3/4 on the unit interval, and the colours in (e) correspond to 1/14, 3/14, 5/14, 7/14, 9/14, 11/14 and 13/14. For convenience, the child clusters associated with specific internal nodes in the tree diagrams are numbered. Birth-year distributions of informants in these child clusters appear on the right.

Figure 1 presents the hierarchical structure of pronunciation variation in the LAMSAS data. In Figure 1a, we colour informants on the map by their finest-scale clusters in the tree diagram. In the *NetStruct* colour scheme, informants with more similar colours appear closer in the tree diagram, and those with distinct colours are placed in different branches at relatively high levels in the hierarchy. For example, in Figure 1a, the distant colours purple and yellow belong to different major branches of the hierarchy; informants coloured purple are mostly in the northern part of the Atlantic region and those coloured yellow are mostly in the southern part.

To examine the two major clusters at a finer level of detail, we reapply the colouring, for each cluster assigning the root node the colour corresponding to the midpoint in the unit interval (Figure 1b and c). Within each of the two clusters, finer levels of the hierarchy group together informants who are geographically closer. In the cluster that contains most of the individuals from the more northerly regions (Figure 1b), pronunciation distinctions can be observed in groups corresponding largely to New York and to West Virginia. In the cluster that contains most of the more southerly individuals (Figure 1b), a distinction is noticeable between finer clusters corresponding to North Carolina and to Virginia, with some individuals in both states placed in small clusters.

We repeat the process to examine Figure 1c in even finer detail. This analysis, in Figure 1d and e, illustrates that at lower levels of the hierarchy, clusters are not always associated with geographical features. However, we observe that year of birth is strongly associated with cluster assignment at this local geographic scale (Figure 1d and e). In other words, in some tree branches, clusters within a branch correspond to age structure, rather than to geography.

This analysis highlights that our clustering extracts one set of features from pronunciation variation at high hierarchical levels – geographical variation in informants – and at lower hierarchical levels, it captures other features, such as age structure. The analysis of multiple hierarchical levels assists in the interpretation of the patterns both at the broadest scale as well as at fine-scale levels.

### Variation in folklore motifs across cultures

In the study of folklore and mythology, recurring plot patterns, or ‘motifs’, occur across cultures. Motif variation can provide insight into cross-cultural patterns, including migrations and cultural transmission in relation to ethnolinguistic barriers (Berezkin, 2010; Bortolini et al., 2017; Korotayev et al., 2017; Thuillard et al., 2018). Here we used folklore motifs to analyse cultural variation, identifying motifs important in constructing the proposed hierarchical relationships.

#### Database of folklore

We examined data on the presence and absence of folklore motifs in individual cultures. Using folklore data from around the world, Berezkin et al. (Berezkin et al., 2009; Korotayev et al., 2017) tabulated recurring motifs prominent in links between folklore traditions, defining a motif to be ‘any image, compositional structure, episode or chain of episodes found in more than one text’. Berezkin et al. reported a list of cultures for each motif.

We focused our analysis on the *n* = 65 regions in the Berezkin et al. database and the *m* = 2459 motifs appearing in at least two of these regions. We computed similarities between pairs of regions based on numbers of shared motifs, negatively weighted by motif frequency (see the Methods section).

#### Hierarchical structure of folklore variation: important characters

**Figure 2.**
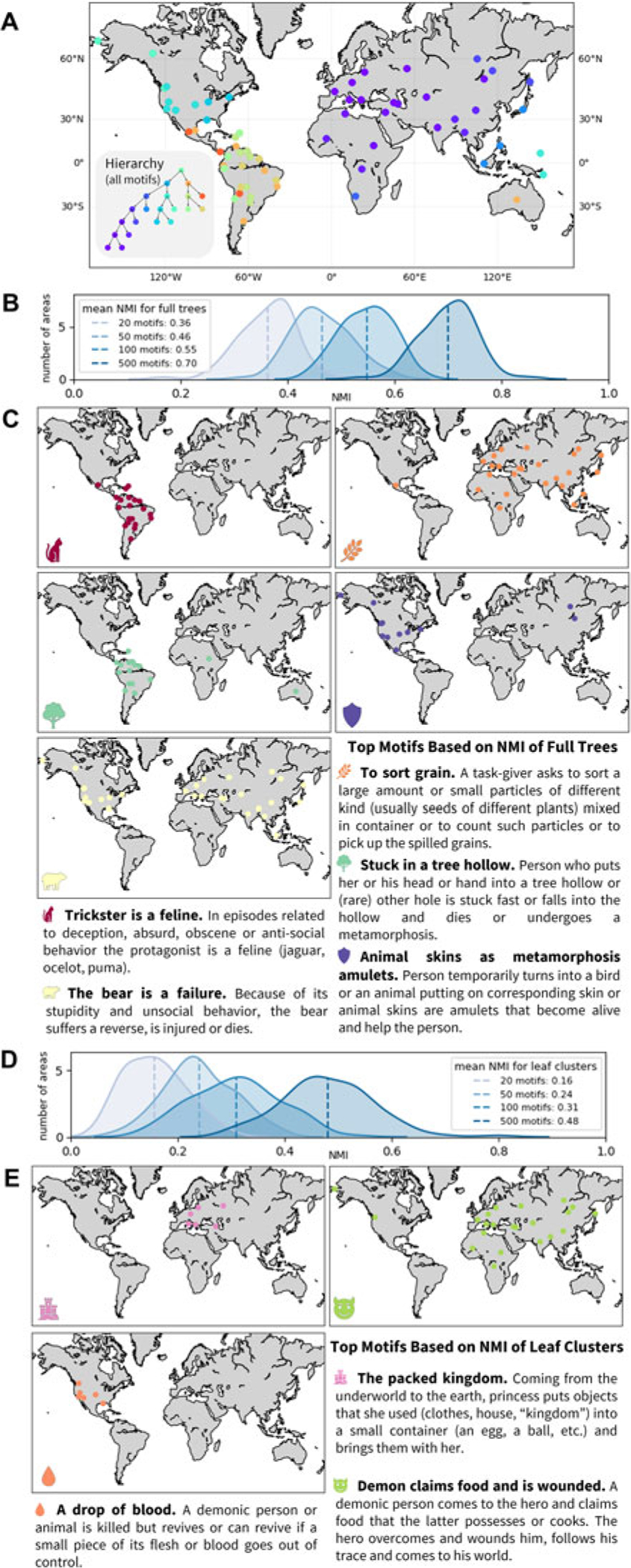
Hierarchical features of variation in folklore motifs across cultures. (a) Hierarchical tree of the motif similarity network. Regions are marked on the map by the colour of the finest-scale cluster to which they belong. (b) Distributions of normalized mutual information (NMI) between hierarchies extracted from sampled subsets of motifs and from all motifs, with 100 subsets of 20, 50, 100 and 500 motifs each. (c) Geographic distributions of five motifs that occur most frequently in the 200 of 5,000 subsets of 20 motifs that produce hierarchies with highest NMI to the hierarchy produced by all motifs. These motifs drive the hierarchy at higher levels, separating regions into major clusters. (d) Distributions of NMI between the leaves of hierarchies extracted from subsets of motifs (those from b) and the leaves extracted from all motifs. (e) Geographic distributions of three motifs that occur most frequently in the 200 subsets that produce hierarchies whose leaf clusters produce highest NMI to those produced by all motifs. These motifs are more specific to the hierarchy in lower levels and potentially capture fine-scale regional differences.

Figure 2a presents the hierarchical structure of motif variation extracted using the pairwise similarities calculated based on all motifs. The geographic regions are mostly clustered into three large areas: Eurasia and Africa (purple), North America (blue) and South America (orange), with varied placement of populations from Australia and Oceania.

To identify which motifs are most important for extracting the hierarchical features, we adopt the normalized mutual information (NMI) approach to compare hierarchies constructed using different sets of motifs (Greenbaum et al., 2019). For a pair of hierarchical clusterings, the NMI measure ranges from 0 to 1, quantifying the information obtained about one clustering by observing another. The NMI measure is high when two clustering hierarchies describe similar clustering structures (see the Methods section). The NMI approach is flexible in that it enables comparisons between subsets of the hierarchical structure, for example by comparing only the leaves of the hierarchy.

We sampled 100 random subsets of 20, 50, 100 and 500 motifs, for each subset applying *NetStruct* to extract a hierarchy from the similarity network based on the sampled motifs. We then computed the NMI between the hierarchy of the sampled motifs and the hierarchy for all motifs, both for the full tree and for only the leaf clusters. In both NMI analyses, as the number of motifs in the subset increases, the mean of the NMI distribution increases (Figure 2b and d). The hierarchy produced by a larger subset of motifs is more informative than those generated with fewer motifs.

Different motifs can be more informative or less informative regarding the hierarchical structure of the data. For example, a motif found in all regions, or one not correlated with the main cultural patterns, will not be informative about the clustering. To identify the most informative motifs, we sampled 5000 subsets of 20 motifs with replacement, counting occurrences of motifs in the 200 subsets possessing the highest NMI with the full tree and those possessing the highest NMI for leaf clusters. With random sampling, the expected number of occurrences of each motif in the top 200 subsets is 20/*m* × 200 ≈ 1.6.

The five most informative motifs for the full hierarchical structure appear in Figure 2c. The motif most frequently found in high-NMI subsets is ‘trickster is a feline’, appearing in 16 of 200 subsets (*p* = 1.5 × 10^−11^, binomial test). This motif is common in Central and South America. ‘To sort grain’ has 11 occurrences (*p* = 9.8 × 10^−7^), and the next three most informative motifs have eight occurrences each (*p* = 2.7 × 10^−4^) and are also associated with large geographic regions (Figure 2c). Some informative motifs correspond to natural or cultural phenomena restricted by geography, such as the practice of agriculture and the habitat ranges of animals.

The three most informative motifs for the fine-scale cultural structure represented by the leaves of the hierarchy are shown in Figure 2e. Each appears eight times, above the number expected from random sampling (*p* = 2.7 × 10^−4^). Two of these, ‘a drop of blood’ and ‘the packed kingdom’, have restricted geographic ranges. This result suggests that motifs of local folklore contribute to fine-scale features of the hierarchy.

In addition to visualizing hierarchical patterns of variation in folklore in relation to geography, this analysis demonstrates the use of *NetStruct* to identify characters – folklore motifs in this case – that play an important role in driving the hierarchical structure. The analysis of many subsets of characters, and the identification of those that appear in subsets that give rise to high NMI with the full-data analysis, uncovers those that contribute most to hierarchical clustering patterns.

### Variation in phonemes across languages

A salient feature of linguistic variation is phonemic variation: variation in the sounds present within languages. Phonemic variation can be used to study inter-language relationships and population migrations (Atkinson, 2011; Creanza et al., 2015; Fort & Pérez-Losada, 2016; Pérez-Losada & Fort, 2018), and for our next example, we analyse hierarchical structure in worldwide phonemic variation.

#### Ruhlen phoneme database

Creanza et al. (2015) analysed two databases that have been assembled on phonemes across large numbers of languages. We applied *NetStruct* on one of these, the Ruhlen database, as studied by Creanza et al. (2015) to explore phoneme-based hierarchical structure across languages. This database contains presence/absence information for 728 phonemes, organized by language classification and geography.

In our analysis, we included all *n* = 2082 languages and *m* = 454 phonemes that exist in more than one language. We then constructed the hierarchy based on the pairwise frequency-weighted phoneme-sharing similarities calculated from the *n* × *m* data matrix (see the Methods section).

#### Hierarchical structure of phonemic variation: Outlier entities

**Figure 3.**
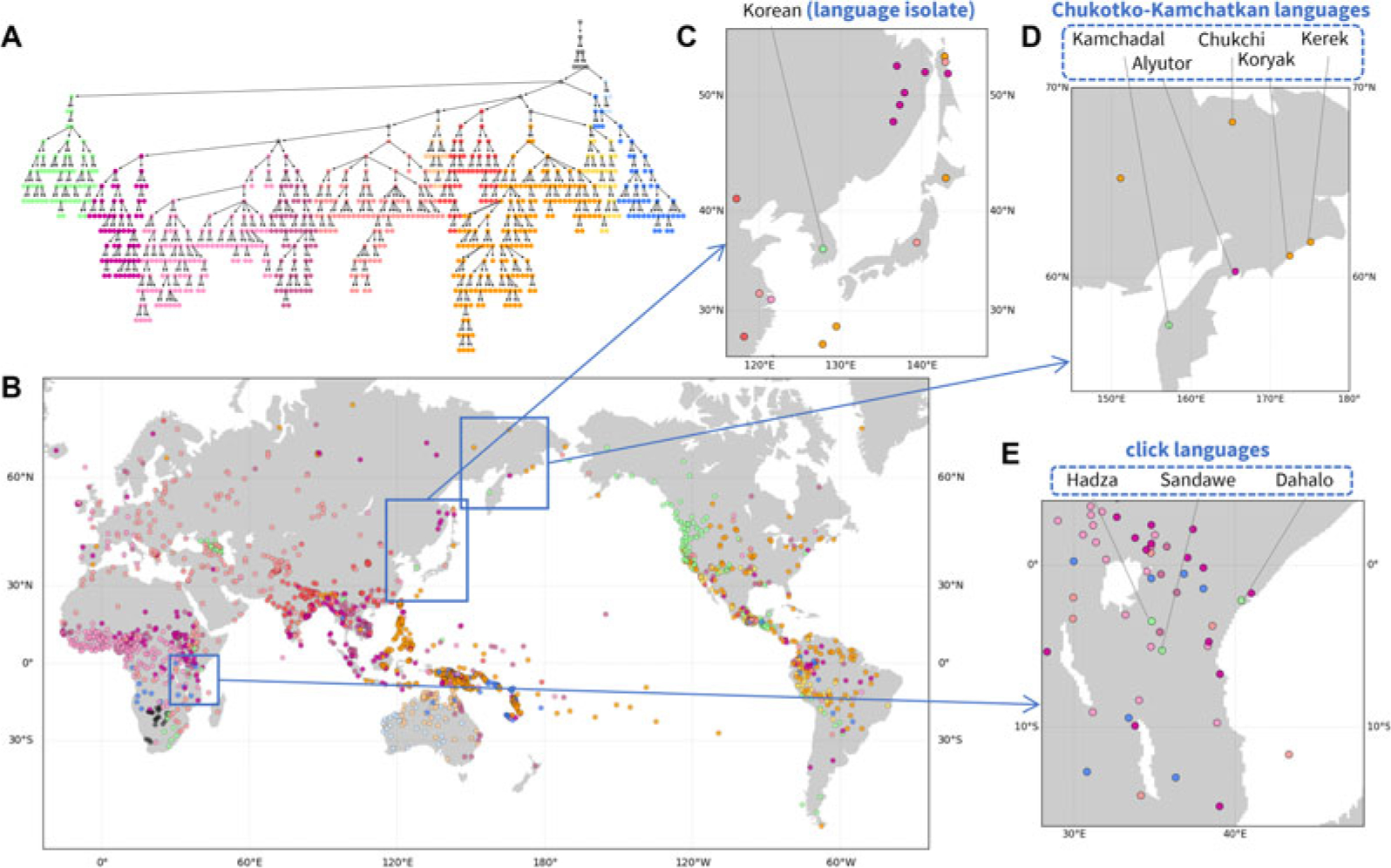
Hierarchical features of phonemic variation. (a) Hierarchical tree of the phoneme similarity network. Major branches that contain most of the languages are assigned distinct colours, and other branches are coloured grey. (b) Language map. Languages are marked by the colour of the finest-scale cluster to which they belong. Three regions are magnified: (c) Northeast Asia; (d) north-eastern Siberia; and (E) East Africa.

The hierarchy extracted from phonemic variation clusters languages in accord with geography on a broad scale (Figure 3a). In Figure 3b, major clusters tend to be localized within continents, in many places co-occurring with other such clusters.

Figure 3c–e highlights patterns in local regions. In northeastern Siberia (Figure 3d), four of five languages of the Chukotko–Kamchatkan language family – Alyutor, Chukchi, Kerek, and Koryak – cluster in one branch (purple to yellow colours), whereas the Kamchadal language is alone in another (green). Indeed, the first four languages and Kamchadal are assigned to different branches in the family, Chukotian and Itelmen, and the unity of the family has been uncertain (Fortescue, 2005).

In East Africa (Figure 3e), three languages shown in a distinct colour from their surrounding languages – Dahalo, Hadza, and Sandawe – are the only three languages in the region that are click languages, a phonemic group of languages for which clicks function as normal consonants (Westphal, 1971). Similarly, in Northeast Asia (Figure 3c), Korean, a language isolate, is clustered into a branch distinct from other neighbouring languages.

This analysis, like the analyses of pronunciation and folklore motifs, illustrates the use of the *NetStruct* framework to identify geographic effects on entities of interest (assemblages of pronunciation variants, folklore motif repertoires and phoneme inventories). The local patterns additionally illustrate the potential of the method for understanding effects on entities – in this case, languages – whose placements in the hierarchy differ from those of their geographic neighbours.

### Variation in first names over time

Frequencies of first names among births in a population represent a rich source of cultural data, enabling tests about mechanisms of cultural change (Hahn & Bentley, 2003; Gureckis & Goldstone, 2009; Berger et al., 2012; Kessler et al., 2012; Acerbi & Bentley, 2014; O’Dwyer & Kandler, 2017). Our final example used *NetStruct* to analyse relationships among names in their patterns of temporal variation.

#### Social Security data on first names

Data on frequent first names from Social Security card applications for births starting in 1880 are provided publicly by the US Social Security Administration. Separately for male and female names, for each year of birth, frequency data are provided. We analysed female and male names separately, restricting attention to 1397 female and 1074 male names of total frequency greater than or equal to 10,000 until the end of 2019.

Considering each year during 1880–2019 separately, the dataset gives two *n* × *m* matrices with *m* = 140 years, and *n* = 1397 for female and *n* = 1074 for male names. The similarity score between two names is computed based on the Pearson correlation between their frequency vectors over the *m* years of available data (see the Methods section). We generated the *NetStruct* hierarchy from these similarities.

To interpret the *NetStruct* hierarchy, we made use of state-specific data, which are available alongside the national data starting from 1910. In the state-level data, each of *n* names has 53 vectors of counts of length 110, for 53 locations (50 states plus District of Columbia, Puerto Rico and other territories) and 110 years (1910–2019). After normalizing counts from each year by the total number of individuals for that year, we identified for each name the state with the greatest mean normalized frequency over 110 years. In other words, we labelled each name by the state in which it was most frequent.

#### Hierarchical structure of variation in temporal patterns among names: Features of entities

**Figure 4.**
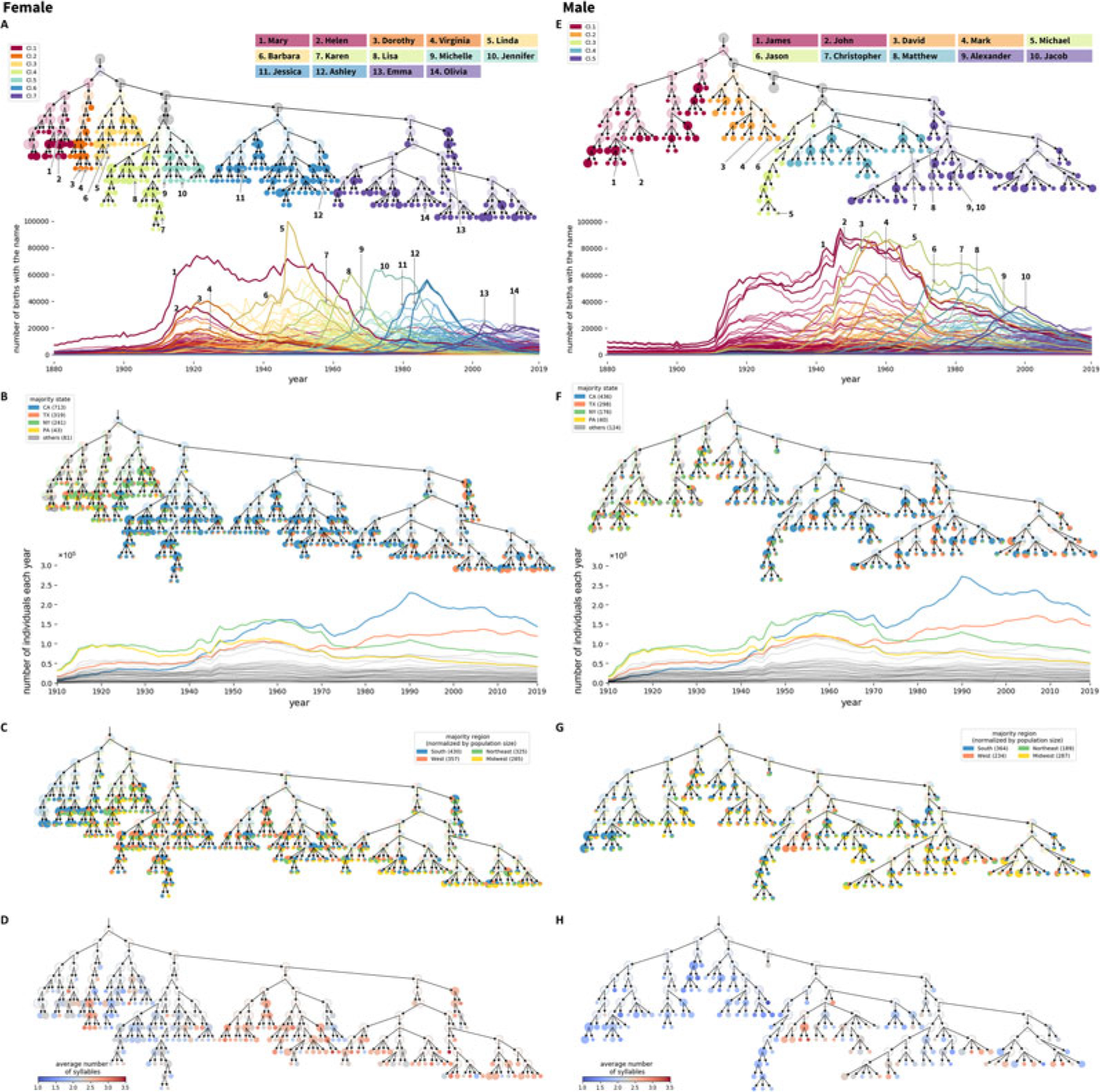
Hierarchical features of time series for frequencies of female names (a–d) and male names (e–h). (a, e) Hierarchical tree of similarity in time series for name frequencies. Major branches are assigned distinct colours. Time series of annual national frequencies appear below the trees, with two names selected from each major branch highlighted. The node area is proportional to the number of names in a cluster, except that clusters containing greater than 25 names are set to a fixed size and are coloured half-transparently. (b, f) Recoding of the hierarchies in (a, e) by states of highest frequency. Each cluster shows a pie chart tabulating the states in which names in the cluster have the highest frequency. Time series of name frequencies appear below the hierarchies. (c, g) Recoding of the hierarchies in (a, e) by regions of highest normalized frequency. The states are grouped into four regions: West (AK, AZ, CA, CO, HI, ID, MT, NM, NV, OR, UT, WA, WY), Midwest (IA, IL, IN, KS, MI, MN, MO, ND, NE, OH, SD, WI), South (AL, AR, DC, DE, FL, GA, KY, LA, MD, MS, NC, OK, SC, TN, TX, VA, WV) and Northeast (CT, MA, ME, NH, NJ, NY, PA, RI, VT). The normalized frequency of a name in a region is the count of the name in the region normalized by the total number of individuals in the region. The steps to obtain the states of highest frequency and regions of highest normalized frequency are described in the Methods section. (d, h) Recoding of the hierarchies in (a, e) by mean number of syllables of names in clusters.

We present the hierarchical structure extracted from time series data on the frequencies of female names, as well as the temporal trends of the corresponding names, in Figure 4a, with seven major branches of the hierarchy coloured differently. The same visualization for male names appears in Figure 4e, with five major branches assigned different colours. For both female and male names, names in branches of different colour have distinct frequency trends over time, with those on the left indicating names that had the greatest frequency at the beginning of the time series.

Figure 4b and f relabel the hierarchies in Figure 4a and e by the state in which a name has occurred most frequently over the full dataset. The calculation of the state with highest frequency for a name is described in the Methods section. Initially, the most populous states were New York and Pennsylvania; California and Texas have been most populous more recently. Thus, the leftmost names, frequent early in the period, tend to be associated with New York, and to some extent, Pennsylvania and Texas. Names in the centre are more closely associated with California. Names in the rightmost clusters are associated with California or Texas, whose recent population growth has reduced the difference from California in the number of annual births.

Because the patterns in Figure 4b and f are driven in large part by population sizes of states, we next relabel the hierarchies using a frequency that is normalized by population size. In particular, we group states into four regions – Midwest, South, Northeast and West – normalizing the region-wise count of each name by the total number of individuals in the region. The calculation of the region with the highest normalized frequency for a name is described in the Methods section. Figure 4c and g relabel the hierarchies in Figure 4a and e by the region in which the normalized frequency is greatest. In this relabelling, the South is the region that has the largest number of names associated with it, for both females and males. This pattern is particularly pronounced at the beginning of the time series, during which the South was the region of greatest frequency for large numbers of names.

Figure 4d and h examine the hierarchies in relation to a second variable: the number of syllables in names. As was seen when considering names by the state with highest frequency, much structure is observable with this variable. Female names in clusters 1 and 2 of Figure 4a, which share a common predecessor node as the parent cluster, have similar temporal trends, with a high frequency in the early twentieth century. In a fine-scale analysis, however, they separate into a branch whose names have fewer syllables (cluster 1, e.g. Mary, Helen), and a branch whose names have more syllables (cluster 2, e.g. Dorothy, Virginia). For male names, later names tend to have more syllables than earlier names (Figure 4h).

In summary, the *NetStruct* analysis reveals relationships in co-occurrences of names, identifying names with similar temporal trends. The recoding of clustering hierarchies by additional variables – the state with highest frequency and the number of syllables – illustrates the use of *NetStruct* in understanding attributes that correlate with, and potentially contribute to, relationships among entities. The visualization can potentially suggest analyses of other factors that influence the dynamics, including immigration, regional correlations and differences in naming practices by state over time.

## Discussion

Inspired by the potential of hierarchical clustering analyses to illuminate features of population-genetic variation, we have adapted the network-based clustering framework *NetStruct* for use in the analysis of cultural variation. In four examples, we have illustrated several aspects of the framework in applications to data matrices representing a set of entities, each associated with values of a set of characters. These applications demonstrate the potential of *NetStruct* to extract broad- and fine-scale relationships among entities. They illustrate the use of *NetStruct* to analyse relationships of geography with clustering patterns, to uncover the characters that drive relationships and to understand effects on cultural data points of interest in specific scenarios. The algorithmic perspective incorporates flexibility in the design of similarity measures and in visualization schemes to aid the analysis.

### Interpretations of data on cultural variation

The four examples illustrate the potential of *NetStruct* for producing novel visualizations to deepen the understanding of cultural entities – pronunciation repertoires of individuals, folklore repertoires of cultures, phoneme inventories of languages and time series of name frequencies. The technique can uncover hierarchical features underlying the variation in cultural traits at different scales, and it enables the examination of different hierarchical levels. For example, the LAMSAS data, the dataset among the four that has been studied for longest, has given rise to numerous analyses of dialect variation, often seeking to partition the Atlantic region into dialects (Lee & Kretzschmar, 1993; Nerbonne & Kretzschmar, 2003; Nerbonne 2015); our approach contributes to observing hierarchical divisions at multiple levels, to detecting spatially continuous variation beyond the level of dialects and to identifying birth date as a variable that contributes to deviations from spatial patterns.

Fewer studies have examined the folklore dataset that we have considered. With the use of NMI, we have shown that *NetStruct* can help to identify informative motifs for describing broad- and fine-scale structures of folklore variation. The recurrence of a shared motif in widely separated cultural groups has been useful for reconstructing cross-cultural contact and examining cultural diffusion. In this context, past studies have considered the diffusion of specific motifs, sometimes chosen as those that are widespread or that have particular cultural salience (Korotaev et al., 2006; Berezkin, 2010; Ross et al., 2013; Tehrani, 2013). Rather than choosing motifs based on prior significance, the NMI approach identifies motifs that are most informative about cultural groupings from patterns of motif occurrence alone. The identification using NMI of motifs of particular informativeness can further focus the choice of specific motifs for use in detailed analysis of diffusion patterns of folklore across worldwide cultural groups; studies such as those examining ‘The Tale of the Kind and the Unkind Girls’ (Ross et al., 2013) and ‘Little Red Riding Hood’ (Tehrani, 2013) can be informative for interpreting patterns in well-known motifs, but studies of other motifs might be more informative for understanding cultural diffusion.

*NetStruct* requires little prior knowledge of datasets of interest. For the phonemes, as in the principal components analysis of Creanza et al. (2015), *NetStruct* identifies broad-scale geographic differentiation by a method that supposes no prior relationships among entities. Our analysis illustrates the potential to highlight distinctions of certain languages from their neighbours, finding that phonemic distinctiveness can reflect the distinctiveness of one language in relation to others.

For first names, previous studies of the data have examined many aspects, including spatial correlations (Barucca et al., 2015; Pomorski et al., 2016) and phonemic influences (Berger et al., 2012); our analyses of the state of greatest popularity and of patterns in syllables contribute further to understanding patterns in name frequencies. Some studies of naming patterns are model based, assuming factors that drive the variation and incorporating these factors as variables in the models to compare with observed trends (Hahn & Bentley, 2003; Gureckis & Goldstone, 2009; Berger et al., 2012; Kessler et al., 2012; Acerbi & Bentley, 2014; O’Dwyer & Kandler, 2017); our approach can augment such studies by suggesting hypotheses that can be used in evaluating different generative models.

In our choices of examples for application of *NetStruct*, the four datasets had several features in common. First, in each case, entities corresponding to rows of the initial data matrix had a natural set of relationships reflected in the *NetStruct* hierarchy – geographic proximity of informants for the pronunciation data, geographic proximity of cultures for the folklore data, geographic proximity of languages for the phonemic data and proximity in time of the period of greatest popularity for the data on names. Second, additional salient attributes of the entities could be considered – birth dates for pronunciation informants, locally specific components of folklore such as geographically restricted cultural practices and animal ranges, family memberships for languages, and states of greatest popularity and numbers of syllables for names. Additional datasets with spatial structure, temporal structure or both, such as data on attributes of ceramics or other artefacts of material culture, or data on individual variation in word choices or other idiolectal variation, potentially provide natural examples as well. For future datasets, the existence of geographic and temporal structure and the availability of other meaningful attributes on entities of interest can be used to support use of *NetStruct* and to guide interpretation of the results that it produces.

### Limitations and extensions

We have chosen to focus on similarity measures borrowed from genetics in which the sharing of a rare genetic variant between two individuals or populations suggests recent common ancestry. Similarly, for cultural data, in which shared descent is also a salient phenomenon, our use of a frequency-weighted trait-sharing similarity measures presupposes the potential importance of shared rare variants in characterizing relationships between entities. However, the choice of similarity measure occurs prior to the application of *NetStruct*; the emphasis of similarity measures on shared rare variants can therefore be tuned as appropriate to a specific type of data. A possible systematic difference from the genetics context is that fast-evolving cultural data could generate more homoplasy than is seen for genetic markers (Tehrani & Collard, 2002; Haasl & Payseur, 2011), so that a shared rare variant could be less meaningful in cultural data than in genetic data. Distance-based hierarchical clustering studies in genetics have generally identified many shared features in population relationships irrespective of the similarity measure considered, even for fast-evolving genetic markers with significant homoplasy (Takezaki & Nei, 1996). In a preliminary analysis of the choice of similarity measure, considering the LAMSAS data, we see that generally similar patterns are obtained with two additional similarity measures: a measure that is not frequency weighted and a measure designed specifically for linguistic data (Figures S1 and S2). With a specific scientific question and dataset, measures that encode aspects of similarity of greatest interest can be considered, and researchers can employ multiple similarity statistics to identify patterns that are robust and patterns that are distinctive to particular measures.

As in genetic studies that use tree-like models of population relationships, we have assumed that a hierarchical relationship between clusters exists, focusing on transferring the application of a hierarchical clustering method from population-genetic data to data on cultural variation. In cultural data, as is often seen in population-genetic data, the appropriate generative model that underlies the data need not be fully tree like. Studies in population genetics have introduced methods for testing the suitability of evolutionary trees for explaining patterns of genetic variation, a key concept being the ‘treeness’ of the data (Cavalli-Sforza & Piazza, 1975; Patterson et al. 2012; Pickrell & Pritchard 2012). It would be of interest to develop comparable approaches for testing the extent to which a hierarchical structure from *NetStruct* explains cultural variation data; a permutation test of Greenbaum et al. (2016) for significance of clustering in a two-level *NetStruct* hierarchy containing a root and offspring nodes, devised in the population-genetic context, can potentially be adapted for arbitrary hierarchies and applied to data on cultural variation.

### Conclusions: Uses and applications of NetStruct

In population genetics, the interplay of evolutionary processes contributes to producing hierarchical patterns in genetic composition among populations. Similarly, in the study of cultural data, many forces interact to shape hierarchical trait variation. Interpreting the clustering results requires consideration of multiple interacting processes and phenomena, including global and local selection pressures on specific cultural variants (e.g. positive, negative, or balancing), the linkage of multiple variants in ‘cultural complexes’ (similar to genetic linkage) and random drift. As in the study of genetic data, geographic patterns need not uniquely identify the underlying processes; for example, similarly to the phenomenon of convergent evolution in genetic data, convergent evolution of cultural variants (e.g. Tehrani & Collard 2002; Mesoudi et al. 2006; Rogers & Ehrlich 2008) can produce a level of similarity that can be conflated with shared descent. For example, in our phonemic analysis in Figure 3, the potential for rapid change in languages can produce similarity in phonemes of otherwise distant languages. The cluster of languages coloured in light green in Figure 3, which includes languages from sub-Saharan Africa, the Caucasus and western North America, may result from convergent evolution combined with linkage of phoneme complexes that have developed independently. Consideration of the mechanistic processes underlying cultural data while incorporating domain-knowledge specific to datasets of interest is important in interpreting the hierarchical structure generated by *NetStruct*. Because cultural data often possess the type of geographic structure, temporal structure, hierarchical categorization or defining attributes for which *NetStruct* results can be productively interpreted, patterns from *NetStruct* can be informative alongside other statistical methods for assessing specific generative models for cultural data.

As a non-model-based tool, the strength of the method lies in its potential as an exploratory approach for producing informative patterns, patterns that potentially inspire hypotheses about factors that drive the features of cultural variation. We suggest that the use of this exploratory approach should be accompanied by analyses of hypotheses based on additional methods and domain knowledge; analyses of data on variation in artefacts of culture can be productively advanced by adding *NetStruct* to the repertoire of the field of cultural evolution.

## Methods

### Between-informant similarity for LAMSAS pronunciation variation

We obtained the LAMSAS data from the project website (www.lap.uga.edu/Site/LAMSAS.html). To eliminate systematic effects of different interviewers, we considered only informants interviewed by the main interviewer, G. Lowman, who collected the earliest LAMSAS data (Nerbonne & Kleiweg, 2003). We therefore restricted attention to 839 informants interviewed during 1933–1942. Because words chosen for pronunciation differed across interviews, many words only appear in the records of a subset of informants. We considered only words collected for at least 700 informants, resulting in a list of 69 words.

Consider *n* informants and *m* words. Suppose word *j* has *l*_*j*_ distinct transcriptions, counting diacritics. Entry *A*_*ij*_ of the data matrix is a categorical variable that indicates the transcription of word *j* for informant *i*: *A*_*ij*_ ∈ {1, 2, …, *l*_*j*_} if the information of word *j* has been collected from informant *i*, or *A*_*ij*_ = 0 if word *j* is unavailable for informant *i*.

We computed a frequency-weighted transcription-sharing similarity, adapting the allele-sharing similarity for genetic data (Greenbaum et al., 2019). For two informants *i*_1_ and *i*_2_, their frequency-weighted transcription-sharing similarity is calculated as

(1) $$s_{i_{1}i_{2}}=\frac{\sum_{j=1}^{m}\left(1-p_{A_{i_{1}j}}^{j}\right){\text{I}}_{\left(A_{i_{1}j}=A_{i_{2j}}\right)}{\text{I}}_{\left(A_{i_{1}j}\neq 0\wedge A_{i_{2}j}\neq 0\right)}}{\sum_{j=1}^{m}{\text{I}}_{\left(A_{i_{1}j}\neq 0\wedge A_{i_{2}j}\neq 0\right)}},$$

where $p_{A_{ij}}^{j}$ is the frequency of transcription *A*_*ij*_ for word *j*. The indicator function *I*( · ) is 1 if the condition holds, and it is 0 otherwise. The similarity matrix *S* is obtained by normalization:

(2) $$S_{i_{1}i_{2}}=\frac{s_{i_{1}i_{2}}-s_{\min}}{s_{\max}-s_{\min}},$$

where $s_{\min}=\underset{i_{1},i_{2}}{\min}\left\{s_{i_{1}i_{2}}\right\}$ and $s_{\max}=\underset{i_{1},i_{2}}{\max}\left\{s_{i_{1}i_{2}}\right\}$.

In rare instances, two individuals have no shared words with data present. In these cases, we assigned for the similarity score the mean similarity of the remaining pairs.

### Between-region similarity for folklore motif variation

We downloaded the folklore data from the Berezkin et al. database (http://www.ruthenia.ru/folklore/berezkin/). The database provides (in Russian) for each indexed motif, a list of all numbered regions in which the motif is present. Considering all 2495 motifs, we constructed the matrix of presence/absence entries, associating the region and motif names with matrix rows and columns, respectively.

We denote the *n* × *m* matrix by *A*, where *n* = 65 is the number of regions and *m* = 2459 is the number of motifs appearing in at least two regions; *A*_*ij*_ = 1 if motif *j* appears in region *i*, and *A*_*ij*_ = 0 otherwise. The pairwise frequency-weighted motif-sharing similarity for two regions *i*_1_ and *i*_2_ is calculated using a weighted Jaccard distance:

(3) $$s_{i_{1}i_{2}}=\frac{\sum_{j=1}^{m}\left(1-f_{j}\right)A_{i_{1}j}A_{i_{2}j}}{\sum_{j=1}^{m}\left(1-f_{j}\right)I_{\left(A_{i_{1}j}\neq 0\vee A_{i_{2}j}\neq 0\right)}}.$$

The quantity $f_{j}=\frac{1}{n}\sum_{i=1}^{n}A_{ij}$ is the frequency of motif *j* across all regions. Equation 3 places greater weight on contributions of less frequent motifs and less weight on common motifs.

We applied the same normalization from Equation 2 to obtain a normalized similarity matrix that we used in our analysis.

### Normalized mutual information

Denote two hierarchical clusterings on *n* entities by *C*^1^ and *C*^2^. Suppose they partition the same set of entities *E*={*e*_1_,*e*_2_, …, *e*_*n*_} into *k* and *l* clusters $C_{1}^{1},C_{2}^{1}\ldots ,C_{k}^{1}$ and $C_{1}^{2},C_{2}^{2}\ldots ,C_{l}^{2}$, respectively, where $\overset{k}{\underset{j=1}{\cup }}C_{j}^{1}=\overset{\ell }{\underset{j=1}{\cup }}C_{j}^{2}=E$. Note that for each clustering – for example, *C* – the clusters are not necessarily disjoint, so that each *e*_*i*_ can belong to multiple clusters *C*_*j*_; indeed child clusters *C*_*j*′_ are contained in parent clusters *C*_*j*_, or *C*_*j*′_, ⊂ *C*_*j*_. The NMI between these two hierarchical clusterings is then computed from the $C_{j}^{1}$ and $C_{j}^{2}$ following the procedure of Greenbaum et al. (2019). This approach is flexible in the sense that NMI can also be computed for subsets of the clusters in the hierarchy, rather than for the entire set of clusters. To address clustering at the finer scale of the hierarchy, we computed NMI for the set of leaf clusters at the tips of the hierarchy.

### Between-language similarity for phoneme inventories

We obtained phoneme data from the supplement of Creanza et al. (2015). The similarity calculation follows that of the motif-sharing similarity, except that *A* now represents an *n* × *m* matrix of *n* = 2082 languages and *m* = 454 phonemes. Equation 3 gives the similarity between a pair of languages, with *f*_*j*_ denoting the frequency of a phoneme among languages; we normalized the similarity matrix by Equation 2 for our subsequent analysis.

### Between-name similarity for name frequency profiles

We downloaded the name data from https://www.ssa.gov/oact/babynames/limits.html. For the analysis, performed separately for female and male names, a matrix entry *A*_*ij*_ tabulates the number of appearances of name *i* in year *j*, normalized by the total number of individuals in year *j*. We write *A*_*ij*_ = 0 if name *i* is absent during year *j*, or if it is rare enough to have been omitted from the database for privacy reasons (fewer than five appearances nationally). For each pair of rows *i*_1_, *i*_2_ of *A*, we computed the Pearson correlation $r_{i_{1},i_{2}}$ between them, and transformed it to a value in [0,1] by $s_{i_{1},i_{2}}=\left(r_{i_{1},i_{2}}+1\right)/2$.

To obtain syllable counts for individual names, two raters separately assigned the counts, discussing cases of disagreement to assign a number of syllables. We computed averages of the number of syllables for names in specific clusters.

### States of highest frequency and regions of highest normalized frequency for names

Separately for female and male names, let *B*_*ijk*_ denote the number of appearances of name *i* in state *k* in year *j*. The frequency of name *i* in state *k* is calculated as $f_{ik}=\left(\sum_{j}B_{ijk}\right)/\left(\sum_{k^{\prime }}\sum_{j}B_{ijk^{\prime }}\right)$. The state of highest frequency for name *i* is obtained by argmax_*k*_(*f*_*ik*_), which we denote the majority state in Figure 4b and f.

The states are then grouped into four regions as described in Figure 4. For each region *l* containing a group of states, let *C*_*ijl*_ denote the number of appearances of name *i* in region *l* in year *j*, or $C_{ijl}=\sum_{k\in l}B_{ijk}$. For each year *j*, this number of appearances is divided by the total number of individuals in a region to obtain the fraction that it represents of all names in the region during year $C_{ijl}^{*}=C_{ijl}/\sum_{i^{\prime }}C_{i^{\prime }jl}$. Averaging across years, the normalized frequency of name *i* in region *l* is then calculated as $g_{il}=\left(\sum_{j}C_{ijl}^{*}\right)/\left(\sum_{l^{\prime }}\sum_{j}C_{ijl^{\prime }}^{*}\right)$. The region of highest normalized frequency for name *i* is obtained by argmax_*l*_(*g*_*il*_), which we denote the majority region in Figure 4c and g.

## Supplementary Material

1

2

3

## Data Availability

The data that support the findings of this study are available as supplementary files. Functions for processing and visualizing *NetStruct* output are provided, as are example uses of those functions.

## Social media summary:

Network-based clustering reveals structure in cultural variation in pronunciation, folklore, phonemes and first names
