# Supplementary material for: Extracting hierarchical features of cultural variation using network-based clustering
Source: Evol Hum Sci. 2022 May 2;4:e18. doi: 10.1017/ehs.2022.15 (PMC9583705; doi:10.1017/ehs.2022.15)
Supplement: Supplementary file 1 [file S2513843X22000159sup.zip › S2513843X22000159sup001.pdf]

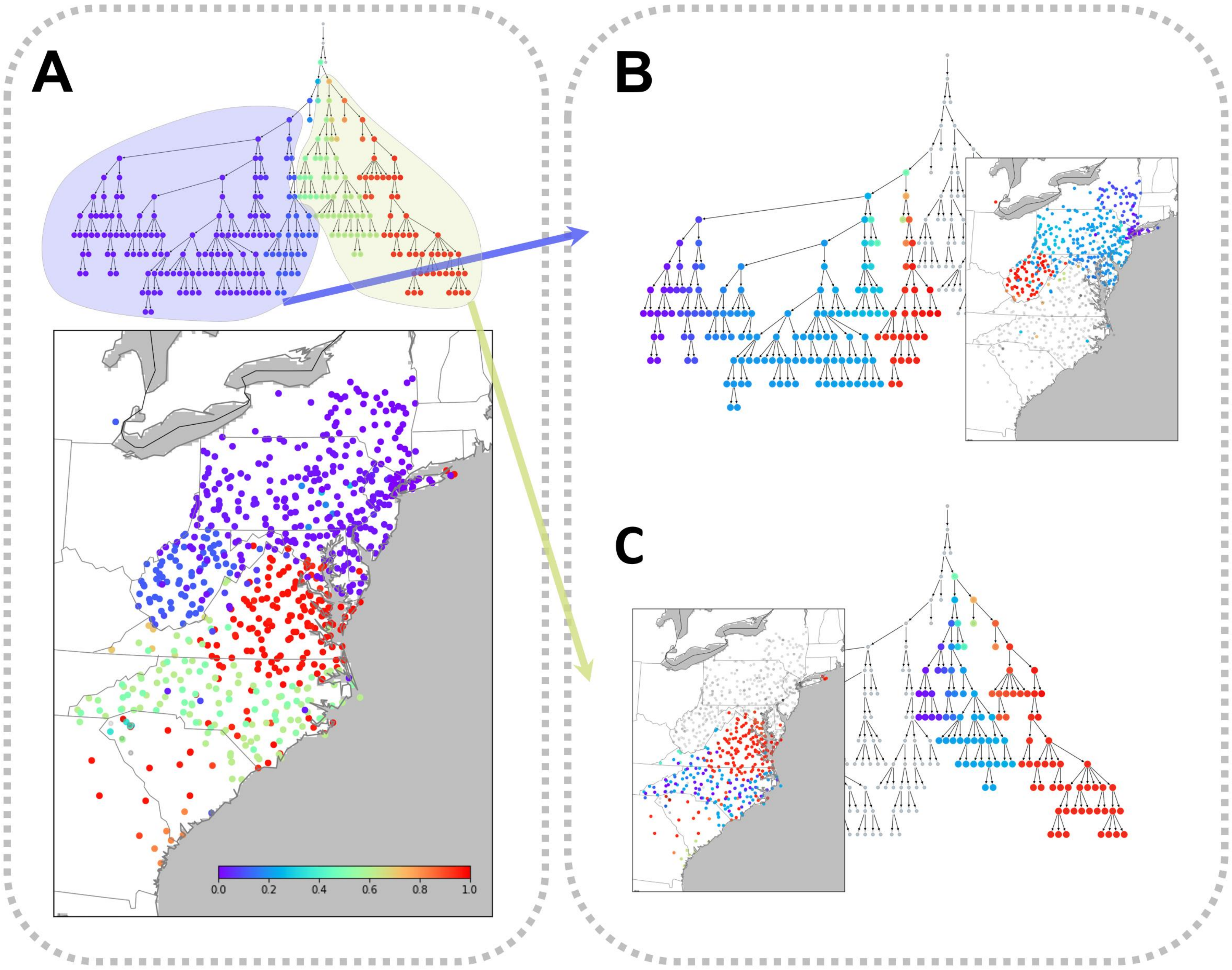

Supplementary Fig. 1: Hierarchical clustering of variation in English pronunciation in the middle and south Atlantic region of the United States, obtained using non-frequency-weighted similarities. (A) Hierarchical tree of the pronunciation similarity network. Informants are marked on the map by the color of the finest-scale cluster to which they belong. (B, C) Two major clusters detected at lower levels of the hierarchy in (A), each re-colored with the full color interval. The figure design follows Figure 1, panels A-C, except that instead of Eq. 1, we compute similarities using the non-frequency-weighted transcription-sharing similarity measure in Eq. S1 (below) before proceeding to Eq. 2. For two informants i_1 and i_2, this similarity is calculated by summing indicator functions that tabulate identity of pronunciations $A_{i_1 j}$ and $A_{i_2 j}$ of word j across the m words:

$$s_{i_1 i_2} = \frac{\sum_{j=1}^{m} I_{(A_{i_1 j} = A_{i_2 j})} I_{(A_{i_1 j} \neq 0 \wedge A_{i_2 j} \neq 0)}}{\sum_{j=1}^{m} I_{(A_{i_1 j} \neq 0 \wedge A_{i_2 j} \neq 0)}}. \qquad \text{(S1)}$$

In the figure, we can see that the hierarchical structure revealed by the non-frequency-weighted similarity is similar to that revealed by the frequency-weighted similarity in Fig. 1; in both cases, two major clusters are split by a north-south divide, and sub-cluster splits also follow geographical lines.

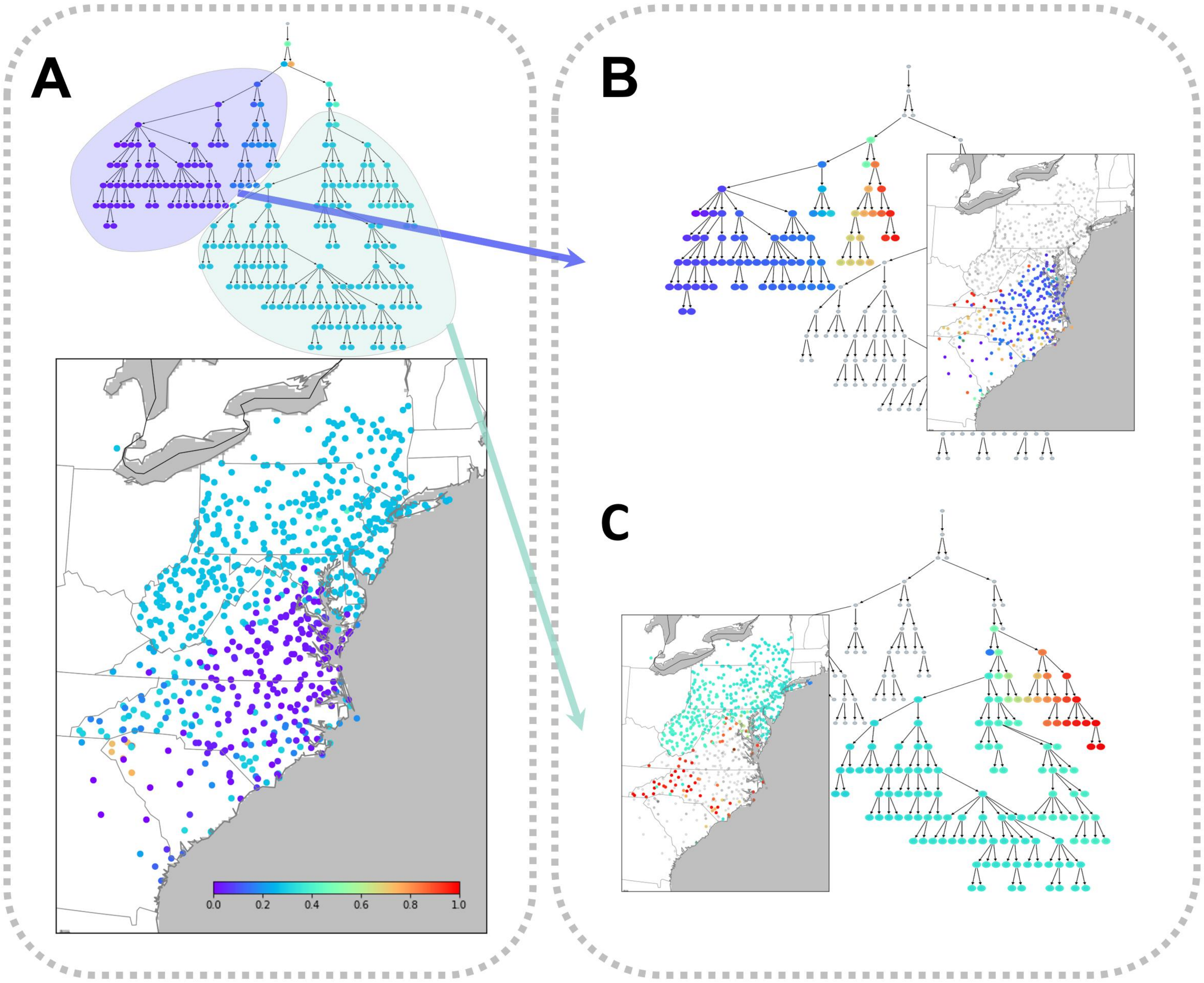

Supplementary Fig. 2: Hierarchical clustering of variation in English pronunciation in the middle and south Atlantic region of the US, obtained using Levenshtein-distance-based similarities. (A) Hierarchical tree of the pronunciation similarity network. Informants are marked on the map by the color of the finest-scale cluster to which they belong. (B, C) Two major clusters detected at lower levels of the hierarchy in (A), each re-colored with the full color interval. The figure design follows Figure 1, panels A-C, except that instead of Eq. 1, we compute similarities using the Levenshtein-distance-based similarity measure in Eq. S2 below before proceeding to Eq. 2. Denote the transcription of word j for informant i as a string T_{ij}. Let $\mathrm{lvd}(\cdot,\cdot)$ be the Levenshtein distance between two transcription strings, computed as the minimum number of single-character edits required to change one string into the other, including insertions, deletions and substitutions (JB Kruskal, An overview of sequence comparison: time warps, string edits, and macromolecules. *SIAM Review* 25: 201-237, 1983). For two informants i_1 and i_2, their Levenshtein-distance-based similarity is calculated as

$$s_{i_1 i_2} = \frac{\sum_{j=1}^{m}\left[1-\frac{\mathrm{lvd}\left(T_{i_1 j},T_{i_2 j}\right)}{\left|T_{i_1 j}\right|+\left|T_{i_2 j}\right|}\right] I_{(A_{i_1 j}\neq 0 \wedge A_{i_2 j}\neq 0)}}{\sum_{j=1}^{m} I_{(A_{i_1 j}\neq 0 \wedge A_{i_2 j}\neq 0)}}, \qquad \text{(S2)}$$

where $|\cdot|$ denotes the length of the transcription string. As in Fig. 1 and Supplementary Fig. 1, the observed hierarchical structure shows a clear multi-level clustering by geographical variation. However, comparing to Fig. 1 and Supplementary Fig. 1, some differences are noticeable. For example, with the Levenshtein distance, West Virginia is separated as a single cluster at a later stage in the hierarchy than for the other two distances. Another difference is that some individuals in the southern region (the red-and-orange branch in Supplementary Fig. 2C) are clustered with the northern region first, and are only later split as a single sub-cluster; in Fig. 1 and Supplementary Fig. 1, they are clustered with the southern part at the first split.
